# Characterisation of the late blight resistance in potato differential Ma*R9* reveals a qualitative resistance gene, *R9a*, residing in a cluster of *Tm*-*2*^*2*^ homologs on chromosome IX

**DOI:** 10.1007/s00122-015-2480-6

**Published:** 2015-03-01

**Authors:** Kwang-Ryong Jo, Richard G. F. Visser, Evert Jacobsen, Jack H. Vossen

**Affiliations:** 1 Wageningen UR Plant Breeding, Wageningen University and Research Centre, P.O. Box 386, 6700 AJ Wageningen, The Netherlands; 2 Graduate School Experimental Plant Sciences, Wageningen, The Netherlands

## Abstract

*****Key message***:**

**The durable late blight resistance in potato plant Ma** ***R9*** **is genetically characterized. A novel** ***R*** **-gene is mapped. The monogenic nature and map positions of** ***R9*** **are negated and rectified.**

**Abstract:**

Late blight of potato (*Solanum tuberosum*), caused by *Phytophthora* *infestans*, can effectively be managed by genetic resistance. The Ma*R9* differential plant provides durable resistance to a broad spectrum of late blight strains. This resistance is brought about by at least seven genes derived from *S. demissum* including *R1*, *Rpi*-*abpt1*, *R3a*, *R3b*, *R4*, *R8* and, so far uncharacterized resistance gene(s). Here we set out to genetically characterize this additional resistance in Ma*R9*. Three BC_1_ populations derived from Ma*R9* were identified that segregated for IPO-C resistance but that lacked *R8*. One BC_1_ population showed a continuous scale of resistance phenotypes, suggesting that multiple quantitative resistance genes were segregating. In two other BC_1_ populations resistance and susceptibility were segregating in a 1:1 ratio, suggesting a single qualitative resistance gene (*R9a*). A chromosome IX PCR marker, 184-81, fully co-segregated with *R9a*. The map position of *R9a* on the distal end of the lower arm of chromosome IX was confirmed using PCR markers GP101 and Stm1021. Successively, cluster-directed profiling (CDP) was carried out, revealing six closely linked markers. CDP^Sw^58, CDP^Sw^59 and CDP^Sw5^10 flanked the *R9a* gene at the distal end (5.8 cM) and, as expected, were highly homologous to *Sw*-*5*. CDP^Tm2^2 flanked *R9a* on the proximal side (2.9 cM). CDP^Tm2^6 and CDP^Tm2^7 fully co-segregated with resistance and had high homology to *Tm*-*2* ^*2*^, showing that *R9a* resides in a cluster of NBS–LRR genes with homology to *Tm*-*2* ^*2*^. Besides R9a, additional resistance of quantitative nature is found in Ma*R9*, which remains to be genetically characterized.

**Electronic supplementary material:**

The online version of this article (doi:10.1007/s00122-015-2480-6) contains supplementary material, which is available to authorized users.

## Introduction

Late blight of potato (*Solanum tuberosum*) is a devastating disease caused by the oomycete pathogen *Phytophthora infestans* (*Pi*) (Fry 2008). This pathogen is a diploid, heterothallic fungus-like oomycete with two mating types (A1 and A2). Over the past several decades, with the increased incidence of late blight epidemics worldwide, the coexistence of both mating types has been observed throughout the world rather than the confinement inside of Mexico (Fry et al. 1993; Goodwin et al. 1994). Consequently, there is a possibility of sexual recombination and increased genetic diversity among progeny of the pathogen which increases the difficulty in late blight control. Furthermore, developing resistance to fungicides in *Pi* populations, which has been demonstrated by widespread resistance to metalaxyl, a key component of fungicides for potato production, requires more frequent applications during the season to control late blight, causing contamination harmful to both humans and environment (Deahl et al. 1993; Goodwin et al. 1994; Grünwald et al. 2001).

Genetic disease resistance has long been considered a promising method for the management of late blight as an alternative to fungicides and healthy seed tubers for late blight control. Two kinds of resistance against late blight have been described in potato: qualitative resistance and quantitative resistance (Rauscher et al. 2010). Late blight qualitative resistance is governed by resistance (*R*) genes that encode immunity through a hypersensitive reaction and is thought to be monogenic, specific, and often of short durability due to the high genetic variability of the pathogen. Since the early part of the last century breeding activities have mainly focused on the high level of resistance conferred by dominant major *R* genes from the Mexican hexaploid wild *Solanum* species, *Solanum demissum.* Presently, many cultivars with *S. demissum* in their pedigree are available (Umaerus et al. 1983; Šwiežynski et al. 1997).

Eleven race-specific *R* genes, named *R1*–*R11*, have been proposed in *S. demissum* and introduced into potatoes (Black 1951; Black et al. 1953; Malcolmson and Black 1966). A current international set of potato *R* gene differentials comprises 11 clones originating from *S. demissum* (Trognitz and Trognitz 2007) for the detection of late blight virulence factors. The Dutch differential set collected by Mastenbroek (1952) is also known as the Mastenbroek differential set: Ma*R1* to Ma*R11*. Ma*R1* to Ma*R4* were developed by Mastenbroek and the other *R* gene differentials are identical to the Scottish differential set developed by Black (Huang 2005). Seven genes controlling late blight resistance within this differential set have been mapped: *R1* on chromosome V (Leonards-Schippers et al. 1992), *R2* on chromosome IV (Li et al. 1998), *R3a*, *R3b*, *R4*, *R6* and *R7* on chromosome XI (El-Kharbotly et al. 1996; Huang et al. 2005; Verzaux 2010) and *R8* on chromosome IX (Jo et al. 2011). Although the differential set was initially thought to represent single late blight resistance factors, many exceptions have been observed: *R1* was also found in the Ma*R5*, Ma*R6* and Ma*R9* differentials (Trognitz and Trognitz 2007) and the Ma*R3* differential plant contained two *R* genes, *R3a* and *R3b* (Huang et al. 2005). Even in the differentials Ma*R8* and Ma*R9*, respectively, at least four (*R3a*, *R3b*, *R4* and *R8*) and seven (*R1*, *Rpi*-*abpt1*, *R3a*, *R3b*, *R4*, *R8* and *R9*) *R* genes were present (Kim et al. 2012). In the past, *R* genes from Ma*R1*, Ma*R2*, Ma*R3*, Ma*R4* and Ma*R10* were rapidly overcome (Wastie 1991), but *S. demissum* is still considered a valuable source for resistance (Niederhauser and Mills 1953; Colon et al. 1995). Especially, the Ma*R8* and *MaR9* have been reported to show broad spectrum resistance both under laboratory and under field conditions (Fry and Goodwin 1997; Šwiežynski et al. 2000; Haynes et al. 2002; Bisognin et al. 2002; Zhang and Kim 2007). Recently, it was shown that this broad spectrum resistance is a result of *R* gene stacking and/or a result of individual broad spectrum *R* genes like *R8*, using a “de-stacking” approach and an “on site” *Pi* virulence monitoring system (Kim et al. 2012). Stacking of multiple late blight *R* genes in different *Solanum* accessions has been revealed (Verzaux 2010) and is most likely a natural defence strategy against the highly flexible late blight pathogen. Also for late blight resistance breeding, stacking of multiple *R* genes seems mandatory to provide sufficient durability (Jo 2013). So far, over 20 functional late blight *R* genes have been cloned and all belong to the CC–NB–LRR class. These include four *Solanum demissum* genes *R1* (Ballvora et al. 2002), *R2* (Lokossou et al. 2009), *R3a* (Huang et al. 2005), and *R3b* (Li et al. 2011) and *R* genes derived from wild *Solanum* species like *S. bulbocastanum* (Song et al. 2003; van der Vossen et al. 2003, 2005; Lokossou et al. 2009), *S. stoloniferum* and *S. papita* (Vleeshouwers et al. 2008), *S. venturii* (Pel et al. 2009; Foster et al. 2009) ,* S. mochiquense* (Jones et al. 2009), *S. chacoense* (Vossen et al. 2010), and *S.* x *edinense* (de Vetten et al 2011).

Quantitative resistance, usually due to the effect of multiple genes of minor effects, is characterized by a slower development of the disease, considered to be race non-specific and influenced by environmental conditions (Wastie 1991). Quantitative resistance locus (QRL) mapping enables to identify multiple loci with important phenotypic effects throughout the genome and characterize epistatic interactions among these loci (Kover and Caicedo 2001). In potato, late blight QRL studies have revealed that some QRLs co-localize with positions of known *R* genes (Stewart et al. 2003; Tan et al. 2008; Rauscher et al. 2010), *R* gene clusters (Gebhardt and Valkonen 2001), and defence genes (Trognitz et al. 2002). Interestingly, some *R* genes were mapped as both qualitative and quantitative traits like *R11* or a quantitative trait like *R10* (Bradshaw et al. 2006). Quantitative resistance has also been reported to be present in plants carrying defeated *R* genes such as *R1*, *R2*, *R10*, and *R11* (Stewart et al. 2003; Pilet et al. 2005) and *Rpi*-*ber* (Rauscher et al. 2010) in the presence of compatible isolates.

In our previous study (Kim et al. 2012), it has been suggested that the Ma*R9* differential contained multiple *R* genes. The present study describes the genetic dissection of the uncovered, remaining resistance from potato differential set plant Ma*R9.* Using a series of BC_1_ populations it was found that the remaining resistance is conferred by a qualitative resistance gene *R9a* as well as by an uncharacterized quantitative resistance. Using a dedicated set of candidate markers, polymorphisms were identified that were linked to, and co-segregated with, *R9a*. It was concluded that *R9a* resides at the bottom end of Chromosome IX, proximal to the *R8* locus. Thereby, the previous suggestion that *R9* would be an allelic variant of *R3* on chromosome XI (Huang et al. 2005) was rejected.

## Materials and methods

### Plant materials

The Ma*R9* differential, corresponding to 2573(2) and LB1 (Black et al. 1953; Malcolmson and Black 1966), was crossed as female parent with the cultivar Concurrent (which contains *R10*). BC_1_ populations were generated by crossing resistant F_1_ progenies as female parents with susceptible cultivar Katahdin as a male parent and six populations were selected (3150, 3151, 3153, 3154, 3155 and 3247). To further characterize 3151, the BC_2_ population 3253 was made by crossing *R8* and *R9a* lacking resistant seedling 3151-03 from BC_1_ population 3151 with cultivar Desiree as a male parent. Neither of the F_1,_ BC_1_ and BC_2_ male parents contains *R* genes that cause incompatibility of the *P. infestans* IPO-C isolate. Seeds were sown under sterile conditions, and plants were maintained in in vitro  culture and propagated for multiple field trials and for the whole plant assays in a climate cell.

### *Phytophthora infestans* isolate and late blight resistance tests

*Phytophthora infestans* isolate IPO-C (race 1, 2, 3a, 3b, 4, 5, 6, 7, 10, 11) was used in both field trials and whole plant climate cell assays. Field trials were done as described by Jo et al. (2011). For whole plant climate cell assays, seedlings from population 3151 and 3154 and 3253 were planted in pots and grown in the greenhouse at 22 °C with a 10 h day/14 h night photoperiod and a relative humidity of 70–80 %. One month after growth of plants, they were transferred into a growth chamber maintained at 15 °C with a photoperiod of 16 h/8 h day/night at 70 % relative humidity. Inoculum preparation and inoculation were performed essentially as described by Vleeshouwers et al. (1999). Three leaves per plant for two plants of each genotype of the populations were inoculated with 10 µl droplets of inoculum (5 × 10^4^ zoospores/ml). 100 % humidity was maintained for the first 3 days after inoculation, after which a humidity of 70 % was restored. Seven days after inoculation phenotypes were classified into three groups, resistant (no symptoms, hypersensitive (HR) lesions), susceptible (sporulating lesions on all inoculated leaves), or intermediate phenotypes. For 3151 and 3154 genotypes that were tested both in the climate cell and in the field trial, there was a complete agreement between field trial and climate cell late blight trials. In the field, scoring was performed by estimating the percentage of blight-affected leaf area.

### Agroinfiltration assay

Two leaves per plant for three plants of each of the BC_1_ genotypes were infiltrated with the following constructs: *Avr8*, 1:1 mixture of *R3a* and *Avr3a* (Bos et al. 2006) as the positive control, and empty pK7FWG2.0 (Karimi et al. 2002) as the negative control. Agroinfiltration assay was carried out as described by Rietman et al. (2012). *Agrobacterium tumefaciens* strain AGL1 + pVirG was used as a carrier of the binary plasmids. *A. tumefaciens* strains were maintained as glycerol stocks and were grown in LB medium supplemented with appropriate antibiotics (*R3a* construct; 50 mg/L kanamycin and 75 mg/L chloramphenicol, *Avr8* and *Avr3a* construct; 50 mg/L carbenicilin and 75 mg/L chloramphenicol) at 28 °C overnight. One day before agroinfiltration, the cultures were reinoculated in YEB medium (5 g beef extract, 5 g bacteriological peptone, 5 g sucrose, 1 g yeast extract, 2 ml 1 M MgSO_4_ in 1 litre of milli-Q water) supplemented with antibiotics, 10 µl of 200 mM acetosyringone and 1000 µl of 1 M MES pH = 5.5. On the day of agroinfiltration, the cells were harvested and resuspended in MMA solution (20 g sucrose, 5 g MS salts and 1.95 g MES in 1 litre of distilled water, adjusted to pH5.5) supplemented with 1 ml of 200 mM acetosyringone to a final OD_600_ of 0.3. The leaves of 4- to 5-week-old potato plants were infiltrated with this suspension. Responses were scored 3–4 days after infiltration.

### DNA isolation and marker analysis

Total genomic DNA was isolated from young leaves as described by Fulton et al. (1995). The Retsch machine (RETSCH Inc., Hannover, Germany) was used to grind young plant materials frozen in liquid nitrogen. For the identification of *R* gene-specific clusters, a modification of the NBS profiling protocol of van der Linden et al. (2004) was carried out as described by Jo et al. (2011). The restriction ligation reaction was done using *Mse*I restriction enzyme and eight *Tm*-*2* ^2^ primers (Tm1R, Tm2F, Tm3F, Tm3R, Tm6F, Tm15F, Tm15R, and Tm19F) described by Verzaux (2010) were used for the successive PCRs. For *Sw*-*5*-CDP, seven specific primers were designed on cluster-specific conserved domains encoding CC and LRR after the alignment of *Sw*-*5* sequences available from NCBI (http://www.ncbi.nlm.nih.gov/). The *Sw*-*5*-specific primers described by Dianese et al. (2010) were also used for *Sw*-*5*-CDP. The CDP primers were used in combination with a labelled adapter primer (fluorescent dye IRD700) and labelled *R* gene-targeted PCR products were separated on a denaturing polyacrylamide gel using a NEN^®^ IR^2^ DNA analyser (LI-COR^®^ Biosciences, Lincoln, NE, USA). A set of the ten resistant and the ten susceptible BC_1_ plants, including parents, was used to obtain CDP markers linked to resistance in mapping population (Vossen et al. 2013). If linked CDP markers were found, a second round of CDP was applied to all the remaining individuals of a segregating population. Fragments were excised as described in the Odyssey^®^ manual for band extraction (Westburg, The Netherlands) and re-amplified with the specific profiling primer and the adaptor primer. The sizes of PCR products were checked on polyacrylamide gels and fragments were cloned into the pGEM-T Easy vector (Promega, USA) prior to sequencing with M13 primers. Sequencing was carried out with the BigDye Terminator kit and an ABI 3700 automated sequencer from Applied Biosystems (USA). The marker nomenclature followed as described by Jo et al. (2011). PCRs for GP101, 184-81, and Stm1021 were performed using DreamTaq^TM^ polymerase (Fermentas) in a standard PCR program (94 °C for 60 s followed by 30 cycles of 94 °C for 30 s, 58 °C for 60 s, 72 °C for 90 s and a final extension time of 5 min at 72 °C). To screen for cleaved amplified polymorphic sequences (CAPS), PCR was done using primers listed in Table 1 and subsequently PCR products were digested using the restriction enzymes listed in Table 1. For SSR, a labelled forward primer (fluorescent dye IRD800) was used to enable visualization on a denaturing polyacrylamide gel using a NEN^®^ IR^2^ DNA analyser (LI-COR^®^ Biosciences, Lincoln, NE, USA). *Tm*-*2* ^*2*^-like or *Sw*-*5*-like sequences available from NCBI (http://www.ncbi.nlm.nih.gov/) and *S. tuberosum Group Phureja* DM1-3 516R44 (CIP801092) Genome Annotation v3.4 (based on v3 superscaffolds) PGSC_DM_v3.4_gene.fasta (http://potatogenomics.plantbiology.msu.edu/blast.html) available from the Potato Genome Sequencing Consortium (PGSC), were collected and aligned using DNASTAR SeqMan Pro™ (DNASTAR. Inc.).

**Table 1:** Markers and primers used in this study

Marker	Primer name	Sequence (5′ → 3′)	Marker type	Tm (°C)	Product size (bp)	References
CDP^Hero3^3	Hero4064F	RRAGATTCAGCCATKGARATTAAGAAA	CDP/*Hae*III	55	500	Jo et al. (2011)
CDP^Tm2^2	Tm19F	GCCAAATAGTATTGTCAAGCTC	CDP/*Mse*I	55	120	Jo et al. (2011)
CDP^Tm2^6	Tm1R	CATTTCTCTCTGGAGCCAATC	CDP/*Mse*I	55	375	Verzaux (2010)
CDP^Tm2^7	Tm2F	CAAGTTTGTCGCAGAGATTGA	CDP/*Mse*I	55	430	Verzaux (2010)
CDP^Sw5^8	Sw3856F	AAGGATGCGACCGTATTGACCTCAT	CDP/*Mse*I	55	118	This study
CDP^Sw5^9	Sw3856F	AAGGATGCGACCGTATTGACCTCAT	CDP/*Mse*I	55	237	This study
CDP^Sw5^10	Sw3856F	AAGGATGCGACCGTATTGACCTCAT	CDP/*Mse*I	55	277	This study
184-81	184-81F	CCACCGTATGCTCCGCCGTC	CAPS/*Rsa*I	58	480	Jo et al. (2011)
	184-81R	GTTCCACTTAGCCTTGTCTTGCTCA				
GP101	GP101F	GGCATTTCTATGGTATCAGAG	CAPS*/Bsp*LI	58	750	GABI
	GP101R	GCTTAACATGCAAAGGTTAAA				
Stm1021	Stm1021F	GGAGTCAAAGTTTGCTCACATC	SSR	58	210	Collins et al. (1999)
	Stm1021R	CACCCTCAACCCCCATATC				
Adapter	Top	CCCGAAAGTATAGATCCCAT			van der Linden et al. (2004)	
	Bottom	TAATGGGATCTATACTT				
Adapter primer		ACTCGATTCTCAACCCGAAAG			van der Linden et al. (2004)	

### Map construction and comparison

Co-segregating, simplex-inherited CDP markers from the tetraploid female parent (Ma*R9*) were scored as dominant markers (Wu et al. 1992). The marker order was determined by TetraploidMap (Hackett and Luo 2003). The map distance was calculated based on the frequency of the recombination between markers. Publicly available potato and tomato genetic maps from SGN (http://sgn.cornell.edu/cview/map.pl?map_id=9&show_offsets=1&show_ruler=1) and GABI (http://www.gabipd.org/database/) databases, and physical map (Jupe et al. 2012) were included for comparison of marker positions and synteny.

## Results

### Development of populations lacking R8

Six IPO-C-resistant F_1_ plants derived from a cross between Ma*R9* and cv. Concurrent (population 3025) were selected in late blight field trials in 2009. To generate BC_1_ populations with a reduced *R* gene content, the selected resistant F_1_ plants were crossed with susceptible cultivar Katahdin. The BC_1_ populations were tested in field trials inoculated with IPO-C in the potato-growing seasons of 2010, 2011 and 2013. Four BC_1_ populations showed a clear segregation into resistant and susceptible groups, while one BC_1_ population (3247) displayed a continuous distribution of late blight severity (Supplementary Figure 1) and could be roughly grouped into three resistant, 37 susceptible and 10 intermediate-resistant individuals (Table 2). To select BC_1_ populations without *R8* the populations were tested for the presence of a *R8* PCR marker (CDP3, which fully co-segregated with resistance; Jo et al. 2011). The progeny of three populations (3247, 3151 and 3154) lacked the CDP3 marker. To rule out that the absence of the CDP3 marker was caused by genetic recombination between *R8* and CDP3, the absence of *R8* was further confirmed by testing the response of the BC_1_ plants to AVR8, the cognate effector of *R8* (Jo 2013). As expected, none of the BC_1_ progeny produced a hypersensitive response (HR) upon AVR8 infiltration (Table 2). According to the marker analysis and effector responsiveness, it was concluded that these BC_1_ populations (3247, 3151 and 3154) did not contain *R8* and that the resistance in these plants must be conferred by additional resistance gene(s). Since the segregation of resistance in population 3247 was of quantitative nature, and because the resistance in populations 3151 and 3154 was of qualitative nature, it was postulated that the remaining resistance in Ma*R9* was constituted by different types of resistances. To avoid suggestions about a potential monogenic nature, we will no longer use the name “*R9”* but refer to the individual genetic components as *R9a*, *R9b*, etc.

**Table 2:** Segregation of late blight resistance, AVR8 response and PCR markers in BC_1_ populations

Population	Segregation of IPO-C resistance^a^	Segregation *R8* marker (CDP^Hero^3)^b^	Segregation of AVR8 response	Segregation *R9a* marker (CDP^Tm2^7)^b^	Resistance caused by
3150	4:6:0	4:6	4:6	Absent	*R8*
3151	8:7:1	Absent	Absent	7:9	*R9a* + QRL
3153	12:5:0	8:9	Not tested	7:10	*R8* and *R9a*
3154	6:4:0	Absent	Absent	6:4	*R9a*
3155	6:9:0	6:9	Not tested	Absent	*R8*
3247	3:37:10	Absent	Absent	Absent	QRL

^a^Number of resistant plants:number of susceptible plants:number of plants with intermediate resistance levels

^b^Number of plants containing the marker:number of plants lacking the marker

### Characterisation of the qualitative* R9a* resistance

Seventeen and ten individuals, respectively, of populations 3151 and 3154 were tested in field trials in 2010 and 2011 and a clear segregation into resistant and susceptible groups was found (Table 2). For mapping the IPO-C resistance a larger population needed to be phenotyped. Since field trials can only be performed in summer, we developed a whole plant late blight assay in a climate cell. The initial small populations that were tested in field trials in 2010 and 2011 were now tested in this climate cell assay and the results fully matched the results of the field assay. In the same climate cell assay 69 additional seedlings of the 3154 population were phenotyped and again a clear segregation into resistant and susceptible groups was observed. In population 3154 resistance segregated in a 1:1 fashion (*χ* ^2^ = 0.5, *p* > 0.05), indicating the expected simplex-based inheritance (Table 3). The causal resistance gene was designated as *R9a*.

**Table 3:** Segregation of late blight resistance and *R9a* marker in two BC_1_ populations

Population (# individuals)	IPO-C phenotype	*R9a* marker^a^ present	*R9a* marker^a^ absent
3154 (*n* = 69)	Resistant	32	0
	Susceptible	0	37
3151 (*n* = 104)	Resistant	47	6
	Susceptible	0	51

^a^PCR marker 184-81 was used

**Fig. 1:**
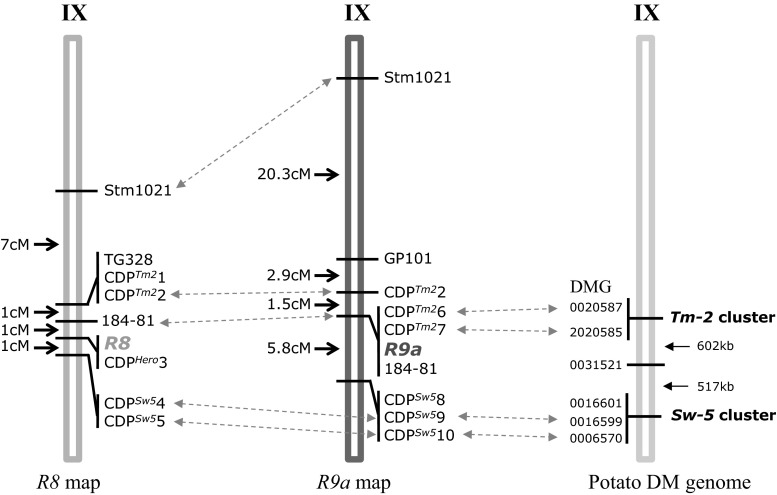
CDP profiles for ten resistant clones and ten susceptible clones of BC_1_ population 3154. *Pr*-resistant parent (Ma*R9*), *Ps*-susceptible parent (Katahdin), *M* molecular weight marker. The *arrows in blue* and *in black* on the *right side* indicate linked markers and the bands size of molecular weight marker, respectively (color figure online)

**Fig. 2:**
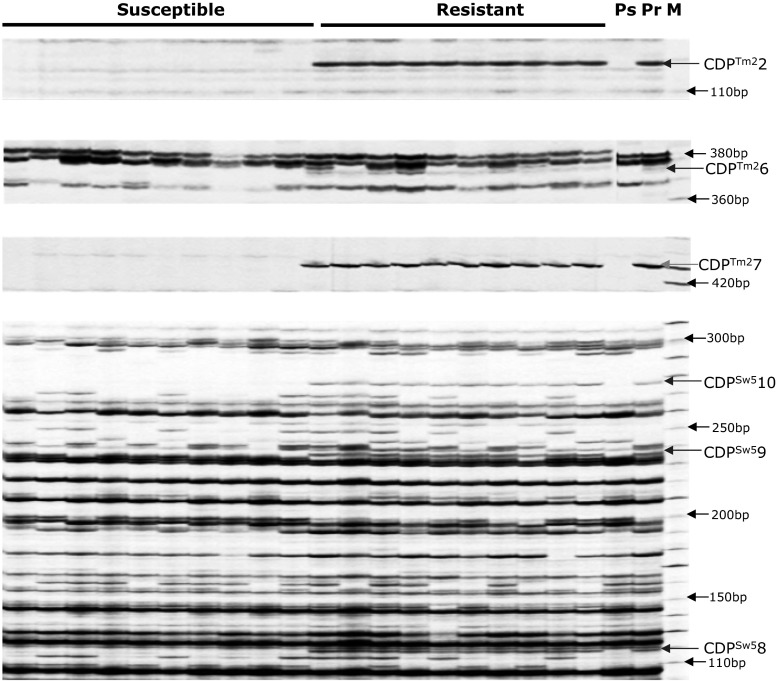
Comparison of *R9a* map with *R8* map and potato DM genome in the distal end of potato chromosome IX. *Blue dotted arrows* indicate similar or identical markers/sequences in different maps. Genetic distances in centimorgan are indicated by *black arrows*. The *R9a* gene maps proximal to the *R8* locus (color figure online)

As described, *R8* (CDP^Hero^3 marker and AVR8 response) was absent from the 3154 population. Interestingly, when the 184-81 marker that flanked the *R8* gene at 1 cM distance was tested in the 3154 population we found that this marker fully co-segregated with the resistance. This suggests that, like *R8*, *R9a* locates on chromosome IX. To verify this finding, we set out to develop additional commonly used markers (GP101, S2g3, TG591A, GP41, CT220, T0521, S1d11, S1d5-a, T1065, TG328, TG424, *St*_At3g23400) from the SGN and GABI databases on the long arm of chromosome IX. A cleaved amplified polymorphism (CAPS) in GP101 was found and located 2.9 cM proximal (two recombinants) relative to *R9a* in population 3154 (Fig. 1). A polymorphism in SSR marker Stm1021, which is present in RH9 BIN65 of the SH x RH map (Van Os et al. 2006), mapped at 20.3 cM (14 recombinants) proximal to *R9a*. In this interval of chromosome IX, two *R* gene clusters (C42 and C43) are known (Jupe et al. 2012). These clusters were targeted for *R* gene cluster-directed profiling (CDP; Vossen et al. 2013). Using eight *Tm*-*2* ^*2*^ primers, population 3154 was screened for linked markers. Three markers, CDP^Tm2^2 (120 bp), CDP^Tm2^6 (375 bp) and CDP^Tm2^7 (430 bp) were identified (Fig. 2) that mapped in close proximity to *R9a.* CDP^Tm2^2 marker is at 1.5 cM distance (one recombinant), proximal from *R9a* and the other two markers fully co-segregated with the resistance in population 3154 (Figs. 1, 2). Using *Sw*-*5*-CDP, three linked markers were found; CDP^Sw5^8, CDP^Sw5^9 and CDP^Sw5^10 (Fig. 2). All CDP^Sw5^ markers were located at 5.8 cM (4 recombinants) to the opposite side (distal) of the CDP^Tm2^ markers from the *R9a* gene (Fig. 1). The CDP markers were excised from the gel and subjected to sequence analysis. The sequence of CDP^Tm2^2 was identical to CDP^Tm2^2 found in mapping *R8* gene (Jo et al. 2011; Genbank accession number JF317285.1). All three CDP^Tm2^ markers identified showed similarity to *Tm*-*2* ^*2*^ and showed 90–92 % identity with PGSC0003DMG402020585. This is an NB-LRR gene which locates in the *Tm*-*2* ^*2*^-like cluster C42 (Jupe et al. 2012). CDP^Sw5^8, CDP^Sw5^9 and CDP^Sw5^10 were confirmed to be similar to *Sw*-*5*, an *S. lycopersicon* tospovirus resistance gene (Brommonschenkel and Tanksley 1997). When the CDP^Sw5^ markers from the *R9a* and *R8* maps were compared with the physical map of unique DMGs encoding NB–LRR-type proteins (Jupe et al. 2012), they were found in cluster C43. Marker CDP^Sw5^10 in the *R9a* map and CDP^Sw5^4 in *R8* map had 70 and 85 % of identity to DMG400016601, respectively. There is a good agreement between the relative positions of the *Tm*-*2* ^*2*^ and *Sw*-*5* homologous markers identified in the Ma*R9*-derived BC1 population and the *R8* map (Jo et al. 2011) and DMG maps (Fig. 1). In conclusion, *R9a* resides on the telomeric end of the southern arm of chromosome IX and locates in or near a *Tm*-*2* ^*2*^ cluster.

### Additional resistance in *Ma*R9, besides *R9a*, is of quantitative nature

Now that *R9a* markers were available, it was possible to better characterise potential additional resistance from Ma*R9*. In BC_1_ population 3153 there was additional resistance besides *R8* (Table 2) since there were four plants without *R8* that were resistant to IPO-C. Using marker CDP^Tm2^7 it was shown that seven plants, including the four *R8*-free plants from the 3153 populations carried the *R9a* gene. This result showed that *R8* and *R9a* are in similar chromosomal location, but they are not linked on the same haplotype. Besides, it was concluded that no additional resistance was present in 3153.

In population 3151 (*n* = 104), the vast majority of the IPO-C-resistant plants carried the 184-81 marker, showing that *R9a* was the major constituent of resistance in population 3151 (*χ* ^2^ = 0.7, *p* > 0.05). However, six plants were observed which were resistant but had neither *R8*, nor *R9a* marker (Tables 2, 3). To further investigate the additional resistance in population 3151, *R9a*- and *R8*-free plants from this population were crossed with susceptible cultivar Desiree to produce BC_2_ population 3253. Two batches of 100 3253 seedlings were inoculated with IPO-C in climate cells. Like in BC_1_ population 3247 no clear segregation into resistant and susceptible groups was observed (Supplementary Figures 1, 2). It was concluded that the additional resistance in BC_1_ population 3151 was of quantitative nature. As described above, in population 3247 a continuous distribution of late blight severity was observed. Using marker analysis, we indeed found that besides *R8*, *R9a* was absent from this population (Table 2). Quantitative resistance may have been caused by residual effects of defeated *R* genes, like the *R10* gene that was introduced through the susceptible parent Concurrent. In our assays we do not expect that *R10* contributes to resistance since Ma*R10* plants are fully susceptible to IPO-C (data not shown).

Based on the currently tested populations we can conclude that the additional resistance in Ma*R9* is constituted of a qualitative resistance gene *R9a* and quantitative resistance that remain to be characterized.

## Discussion

### Characterization of the *Ma*R9 resistance

In this study, we genetically characterized the additional resistance to *P. infestans* isolate IPO-C from the late blight differential plant Ma*R9*. Both qualitative and quantitative resistances were encountered. The qualitatively inherited resistance gene *R9a* resides on the distal end of chromosome IX like *R8*. Both *R8* and *R9* genes have previously been suggested to locate on chromosome XI as allelic variants of *R3* (Huang et al. 2005) because of the presence of the *R3a* haplotype in the *R8* and *R9* plants. In retrospect, the presence of the haplotype was associated with the presence of the *R3a* gene itself that was a contaminant of the Ma*R8* and Ma*R9* differentials (Kim et al. 2012) rather than with the location of the *R8* and *R9a* genes in the *R3* haplotype. We employed an *R* gene “de-stacking” (making offspring plants containing different *R* gene combinations) approach using marker analysis and effector response to eliminate the effects of other known *R* genes for revealing the remaining resistance in Ma*R9*. The resulting BC_1_ populations were analysed using *R* gene cluster-directed profiling (CDP) strategies for mapping *R9a*. It was essential to make a clear discrimination between *R9a* and *R8* because Ma*R9* contained *R8* as well as *R9a* and because isolate IPO-C was avirulent on both *R8*- and *R9a*-containing plants. Analysis of the absence of *R8* in mapping populations using the *R8* marker alone was not sufficient since *R9a* was in a similar chromosomal position as *R8.* Another molecular tool, *in planta* AVR8 expression, was used to further confirm the absence of *R8*. It was confirmed that populations lacking the *R8* marker indeed were not responsive to AVR8, so it could be concluded that the *R8* gene could not be responsible for the resistance to IPO-C in the selected populations. By investigating BC_1_ and BC_2_ populations, it could also be revealed that quantitative resistance contributes to the resistance to IPO-C in Ma*R9*. Regarding the quantitative resistance, there would be two possibilities: (1) QRL as a new genetic entities and/or (2) residual effects of resistance (Stewart et al. 2003; Rauscher et al. 2010) from different combinations of the defeated *R* genes from Ma*R9*, as well as *R10* from cv. Concurrent when progeny plants were infected with a compatible isolate IPO-C. Further analysis regarding insights into the genetic nature and stability of the quantitative resistance in Ma*R9* through isolates and environments remains to be conducted.

### The long arm of chromosome IX is a hot spot for resistance

The long arm of chromosomes IX is a hot spot for resistance in *Solanaceous* genomes (Sliwka et al. 2006; Pel et al. 2009). The *R* gene clusters on the long arm of chromosome IX contain the late blight resistance genes *Rpi*-*moc1* of *S. mochiquense* (Smilde et al. 2005) *Rpi*-*phu1* which is identical to *Rpi*-*vnt1,* most likely from *S. phureja* (Sliwka et al. 2006), *Ph*-*3*, a major QRL for late blight resistance from *S. pimpenelifolium* (Zhang et al. 2014) the *R8* late blight resistance gene from *S. demissum* (Jo et al. 2011), *Rpi*-*dlc1*, from *S. dulcamara*, a *Solanum* species native to Europe (Golas et al. 2010), and *Rpi*-*edn2* from *S.x edinense*, a natural pentaploid hybrid between *S. demissum* and the South American cultivated potato *S. tuberosum* spp. *Andigena* (Verzaux 2010), mapped in a similar region as *R9a.* Also resistances to other diseases like *Sw*-*5*, a tomato gene for tospoviruses resistance (Brommonschenkel and Tanksley 1997), *Nx* encoding hypersensitive resistance to Potato virus X of *S. phureja* (Tommiska et al. 1998), *Gpa6*, a QRL for resistance to *Globodera pallida* in potato (Rouppe van der Voort et al. 2000), the gene *Gm* conferring resistance to Potato virus M (Marczewski et al. 2006), the *Ry*_chc_ and *Ny-1*  potato virus Y resistance genes (Sato et al. 2006; Szajko et al. 2008), as well as a QRL for resistance to *Erwinia carotovora* ssp. *atroseptica* (Zimnoch-Guzowska et al. 2000) have been identified in similar genomic regions. The long arm of chromosome IX features two large heterogeneous clusters (Jupe et al. 2012). Cluster 42 harbours eight TIR–NB–LRR genes that are separated by eight paralogs of *Tm*-*2* ^*2*^, whereas the distal cluster C43 contains 15 paralogs of the Tospovirus resistance gene *Sw*-*5* (Jupe et al. 2012). When compared to tomato, these clusters seem to have drastically expanded in tomato as both the *Tm*-*2* ^*2*^ and Sw-5 cluster in the Heinz tomato genome count only four members (Andolfo et al. 2014). Alternatively, tomato clusters may have been subject to shrinking as the *S. pimpenellifolium* genome only counts two *Tm*-*2* ^*2*^ paralogs (Zhang et al. 2014). Therefore, *R* genes on the long arm of chromosomes IX could be members of either the *Tm*-*2* ^*2*^ cluster or the *Sw*-*5* cluster so that *R* gene cloning by paralog mining (Vossen et al. 2014) or other homology-based approaches (Jupe et al. 2012 would be encouraged, although it will require sophisticated bioinformatics for efficient assembly of paralogous sequences.

### Implications for late blight resistance breeding

The utility of *R9a* in combination with the *R8* gene would be of particular interest in the context of late blight resistance breeding of potato. The co-introgression of multiple *R* gene(s) is essential to provide durability to new potato varieties. Another candidate for co-introgression is *R2* that is known to be defeated but remains providing quantitative resistance in many potato-growing regions (Pilet et al. 2005; Wang et al. 2012; Li 2012), could most likely contribute to durability. The combination of *R2,* *R8* and *R9a* was correlated with durable resistance observed in Ma*R9* (Kim et al. 2012; Jo 2013). However, *R9* QRL were most likely unevenly distributed over Ma*R9* BC_1_ offspring in these studies. The presence of the *R* gene combinations in dissimilar genetic backgrounds may have blurred this on-site durability monitoring approach. The study of *R* genes and *R* gene combinations in isogenic genetic backgrounds is essential for future durability studies of *R* gene combinations (Zhu et al. 2014).

Two approaches can be used to produce potato clones with late blight *R* gene combinations; marker-assisted selection or GM breeding strategies (Zhu et al. 2013). Marker-assisted selection allows transfer of multiple *R* genes into potato using traditional breeding methods, an alternative to deploying the *R* gene through genetic transformation which has been a non-stop controversial issue. The fact that *R9a* and *R8* genes reside in the adjacent *Tm*-*2* ^*2*^- and/or *Sw*-*5* clusters which are physically separated by a maximum distance of 1120 kb (Fig. 2), and that they are naturally located on different chromatids (this study), provide a potential challenge to recombining them into coupling phase, as demonstrated by Robbins et al. (2010). This would result in a chromosome fragment containing *R8* and *R9a*. As a consequence, possible genes with a negative effect (linkage drag) located between *R8* and *R9a* will not be lost by backcrossing. Cisgenic breeding based on the introduction of cloned natural *R* genes from crossable species using *A. tumefaciens*-mediated marker-free transformation, would not encounter the problem of linkage drag that could not be removed (Jo et al. 2014).

#### **Author contribution statement**

JKR performed the molecular experiments and manuscript writing. RV and EJ were involved in manuscript writing and revisions. JV performed phenotyping experiments and manuscript writing.

## Electronic supplementary material

Below is the link to the electronic supplementary material.

Supplementary material 1 (PPTX 49 kb) Supplementary Fig. 1. Blight scores (mean of four plants) for 51 progeny in BC_1_ population in field trials. BC_1_ population 3247 was generated by crossing resistant F_1_ progeny (3025-2, originating from F_1_ of Ma*R9* x Concurrent) as female parents with susceptible cultivar Katahdin as a male parent. Blight scoring was performed by estimating the percentage of blight-affected leaf area in the field

Supplementary material 2 (PPTX 3383 kb) Supplementary Fig. 2. Late blight symptoms on population 3253 seedlings in climate cell assays. A continuous distribution in resistance/susceptibility levels to IPO-C was noticed
