# Supplementary figures and images for: Characterisation of the late blight resistance in potato differential MaR9 reveals a qualitative resistance gene, R9a, residing in a cluster of Tm-22 homologs on chromosome IX
Source: Theor Appl Genet. 2015 Mar 1;128(5):931–41. doi: 10.1007/s00122-015-2480-6 (PMC4544503; doi:10.1007/s00122-015-2480-6)

## Slide 1
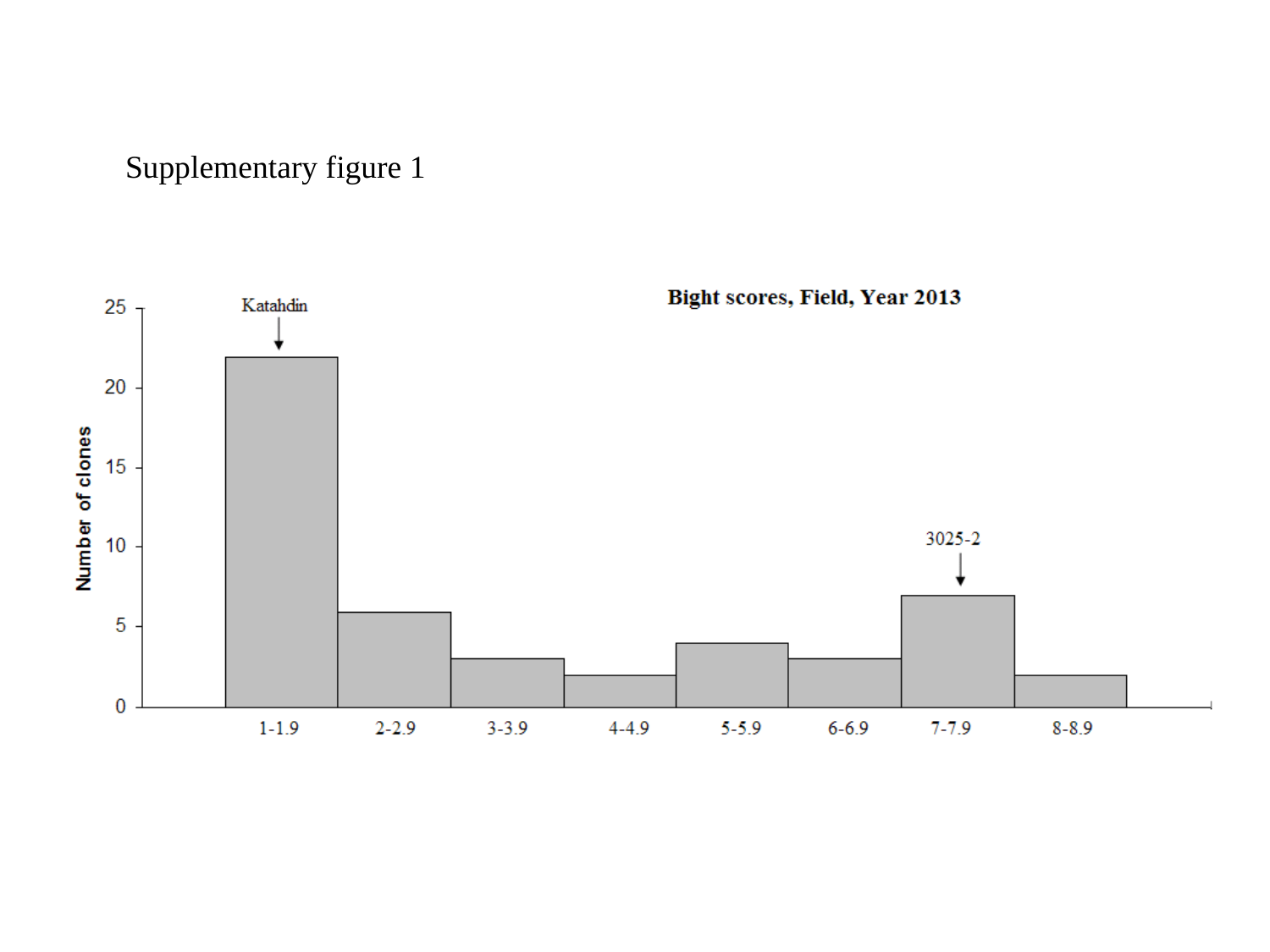

Supplementary figure 1

Supplement: Supplementary file 1 — Supplementary material 1 (PPTX 49 kb) Supplementary Fig. 1. Blight scores (mean of four plants) for 51 progeny in BC1 population in field trials. BC1 population 3247 was generated by crossing resistant F1 progeny (3025-2, originating from F1 of MaR9 x Concurrent) as female parents with susceptible cultivar Katahdin as a male parent. Blight scoring was performed by estimating the percentage of blight-affected leaf area in the field [file 122_2015_2480_MOESM1_ESM.pptx]

## Slide 1
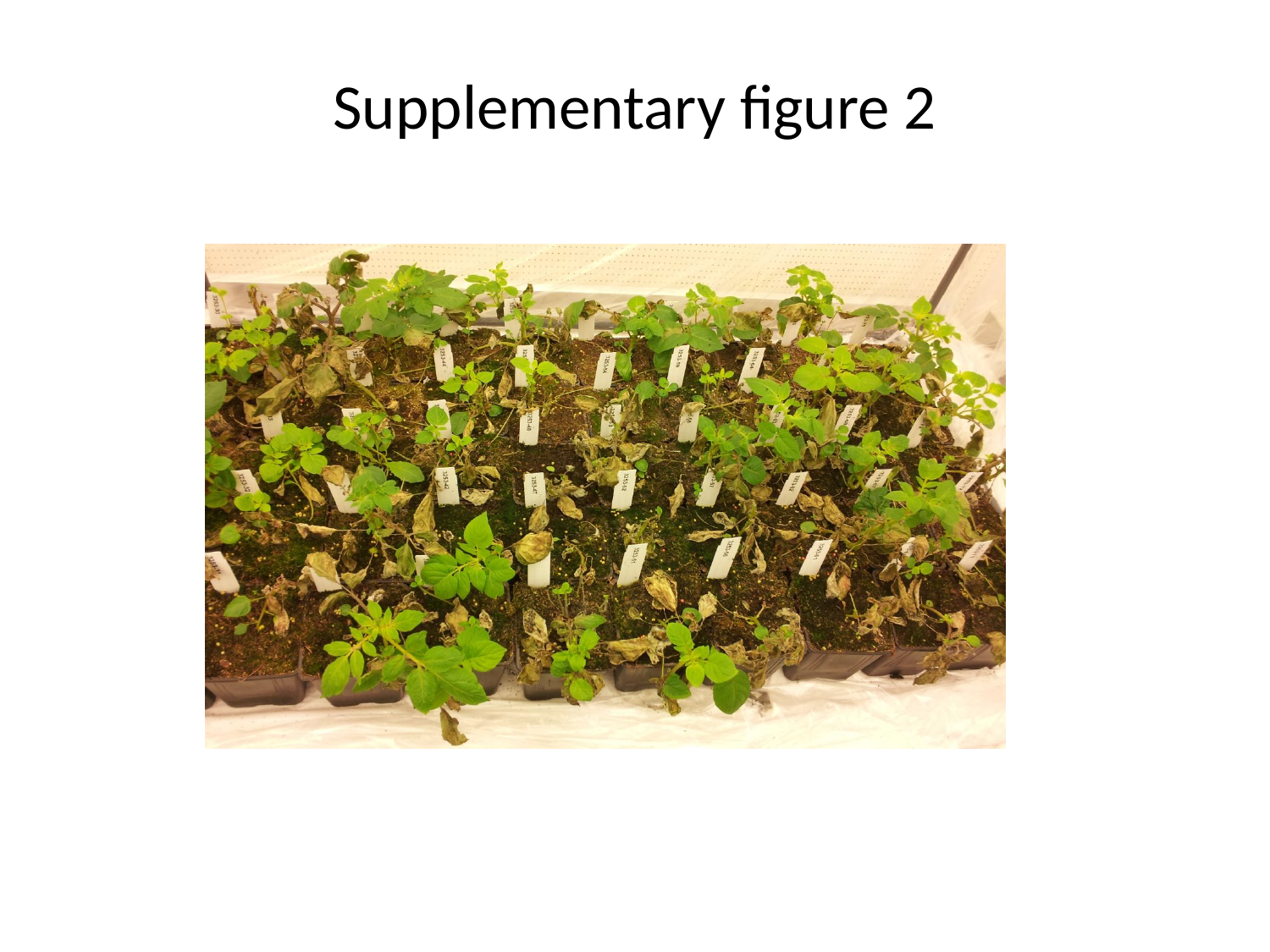

# Supplementary figure 2

Supplement: Supplementary file 2 — Supplementary material 2 (PPTX 3383 kb) Supplementary Fig. 2. Late blight symptoms on population 3253 seedlings in climate cell assays. A continuous distribution in resistance/susceptibility levels to IPO-C was noticed [file 122_2015_2480_MOESM2_ESM.pptx]
